# Serologic Surveillance of Anthrax in the Serengeti Ecosystem, Tanzania, 1996–2009

**DOI:** 10.3201/eid1703.101290

**Published:** 2011-03

**Authors:** Tiziana Lembo, Katie Hampson, Harriet Auty, Cari A. Beesley, Paul Bessell, Craig Packer, Jo Halliday, Robert Fyumagwa, Richard Hoare, Eblate Ernest, Christine Mentzel, Titus Mlengeya, Karen Stamey, Patricia P. Wilkins, Sarah Cleaveland

**Affiliations:** Author affiliations: University of Glasgow, Glasgow, Scotland (T. Lembo, K. Hampson, H. Auty, P. Bessell, S. Cleaveland);; Lincoln Park Zoo, Chicago, Illinois, USA (T. Lembo);; Centers for Disease Control and Prevention, Atlanta, Georgia, USA (C.A. Beesley, K. Stamey, P.P. Wilkins);; University of Minnesota, St. Paul, Minnesota, USA (C. Packer);; University of Edinburgh, Edinburgh, Scotland (J. Halliday);; Tanzania Wildlife Research Institute, Arusha, Tanzania (R. Fyumagwa, R. Hoare, E. Ernest);; Endangered Wildlife Trust, Parkview, South Africa (C. Mentzel);; Tanzania National Parks, Arusha (T. Mlengeya)

**Keywords:** Communicable disease, anthrax, bacteria, Bacillus anthracis, serology, surveillance, environmental exposure, disease susceptibility, Tanzania, research

## Abstract

*Bacillus anthracis*, the bacterium that causes anthrax, is responsible for varying death rates among animal species. Difficulties in case detection, hazardous or inaccessible carcasses, and misdiagnosis hinder surveillance. Using case reports and a new serologic assay that enables multispecies comparisons, we examined exposure to and illness caused by *B. anthracis* in different species in the Serengeti ecosystem in Tanzania during 1996–2009 and the utility of serosurveillance. High seroprevalence among carnivores suggested regular nonfatal exposure. Seropositive wildebeest and buffalo showed that infection was not invariably fatal among herbivores, whereas absence of seropositivity in zebras and frequent detection of fatal cases indicated high susceptibility. Exposure patterns in dogs reflected known patterns of endemicity and provided new information about anthrax in the ecosystem, which indicated the potential of dogs as indicator species. Serosurveillance is a valuable tool for monitoring and detecting anthrax and may shed light on mechanisms responsible for species-specific variability in exposure, susceptibility, and mortality rates.

Anthrax, which is caused by the gram-positive, sporulating bacterium *Bacillus anthracis*, primarily affects herbivorous livestock and wildlife species, but also poses serious public health risks in many parts of the world. Carnivores may also become infected by ingesting contaminated carcasses, but disease-associated illness and death are rarer than in herbivores. Although the multihost nature of the pathogen presents epidemiologic challenges, heterogeneities in host range and infection outcome provide opportunities for disease surveillance, e.g., through the use of sentinel or indicator species to detect the pathogen and changes in its prevalence or incidence (*1**,**2*).

Despite the recognized value of serologic data for disease surveillance and epidemiologic investigations, serologic analysis has only rarely been used in studies of anthrax. One reason may be the perception that case detection is relatively straightforward: a syndrome of sudden death in herbivores is useful for presumptive diagnosis, and microscopic examination of blood smears provides a relatively simple confirmatory test. However, in many environments in which anthrax is endemic, carcasses deteriorate rapidly, are hazardous, and may be inaccessible for sampling for laboratory confirmation. The utility of carcasses for case detection depends on the likelihood of observation and subsequent reporting (*3*). In many parts of Africa, anthrax is typically documented only during large, dramatic outbreaks (*4**–**7*). In remote areas or during small outbreaks, carcasses often go undetected and, even when detected, may provide only an incomplete picture of spatiotemporal patterns of infection. In humans, many anthrax cases are not reflected in hospital records. Underreporting is particularly likely for pulmonary and gastrointestinal anthrax, which have high case-fatality rates and pose diagnostic challenges (*8**,**9*), leading to a lack of appreciation of the true scale of the disease in anthrax-endemic regions.

Another explanation for the lack of serologic studies may be the perception that because sudden death is a distinctive feature of anthrax in herbivores, most infected animals will not survive to produce an antibody response. However, susceptibility varies widely among species, and even within a susceptible species, seropositive animals have been detected, e.g., in cattle in the United Kingdom (*10*) and bison (*Bison bison*) in North America (*11*). Turnbull et al. documented serologic evidence of infection in Etosha lions (*Panthera leo*) and suggested that lions can serve as an indicator species of anthrax in disease-endemic areas because of their territorial behavior (*10**,**12*). The potential of using serologic analysis for comparative studies has also been limited because, up until now, serologic assays for detecting antibodies to anthrax in a variety of species have not been widely available, even in research settings.

The constraints of anthrax surveillance, particularly in tropical areas, highlight the need to identify alternative approaches to overcome these difficulties. We present results of analyses of data obtained opportunistically in the Serengeti ecosystem in Tanzania to explore the value of serologic analysis for providing information about anthrax infection and exposure patterns in large, remote, and complex ecosystems. Using seroprevalence data obtained with 1 assay for a range of species, we also investigate within-species and between-species variations in exposure and survival to evaluate which species may be useful as indicators of anthrax for surveillance purposes, specifically to identify high-risk areas for human and livestock populations.

## Materials and Methods

### Study Area

**Figure 1:**
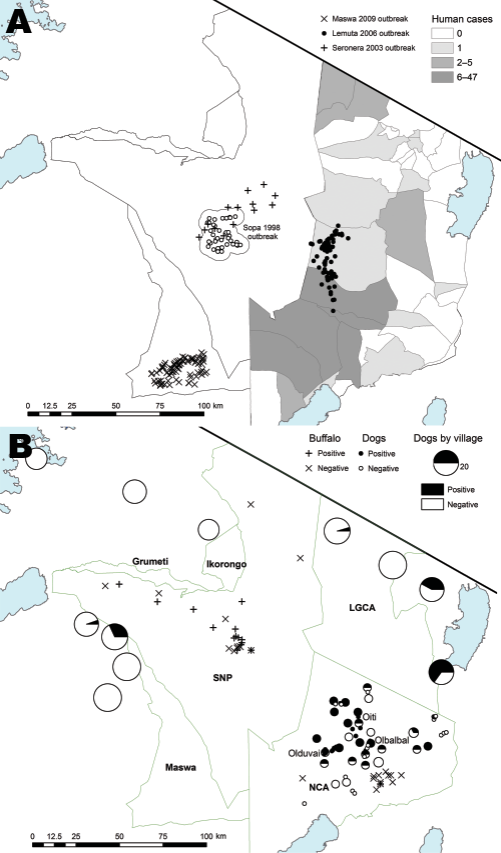
Anthrax cases and exposure to anthrax in the study area, Tanzania. Blue areas indicate lakes. A) Location of wildlife carcasses during anthrax outbreaks. Shaded areas indicate regions where human anthrax cases were reported during 1995–2008. Exact locations of carcasses obtained during the Sopa 1998 outbreak were not available; open circles indicate area where 549 probable cases and 67 suspected cases were detected. For the Seronera 2003 outbreak, locations of cases were randomized within a 10-km radius of the outbreak area because exact locations of carcasses were not available. B) Seroprevalence in domestic dog populations from sampled villages. Sample size is indicated by the radii of the pie charts. Green border indicates Serengeti ecosystem. LGCA, Loliondo Game Control Area; SNP, Serengeti National Park; NCA, Ngorongoro Conservation Area.

The study area in the Serengeti ecologic region in northwestern Tanzania comprised wildlife-protected areas, including the Serengeti National Park (SNP) and adjacent game reserves (Maswa, Ikorongo, and Grumeti). Study sites also included multiethnic, agropastoralist communities west of SNP and Ngorongoro District east of SNP, a multiple land use game controlled area inhabited by low-density Maasai and Sonjo communities that had production systems based on traditional pastoralism and limited cultivation. Ngorongoro district is divided into the Loliondo Game Control Area in the north and the Ngorongoro Conservation Area (NCA) in the south (Figure 1).

### Disease Monitoring Operations

Disease detection in wildlife was based upon passive surveillance operations in the study area during 1996–2009. Sightings of carcasses were reported through a network of veterinarians from Tanzania National Parks (TANAPA) and Tanzania Wildlife Research Institute (TAWIRI), rangers, scientists, and tour operators.

Passive surveillance data from outside SNP were available through veterinary office records (government offices, TAWIRI, TANAPA, and NCA Authority). Human anthrax cases were compiled from records of government and mission hospitals in the study area for 1995–2008. Further information about human anthrax cases was obtained through key informants in villages affected by a major anthrax outbreak in 2006, and data on the age, sex, clinical presentation, and outcome of infection of affected family members was obtained from heads of affected households. No human cases were confirmed by laboratory diagnostic tests.

### Case Definitions

Because of the relatively low proportion of suspected cases from which diagnostic samples were obtained, cases were classified as suspected for carcasses found that had no obvious cause of death, and probable for carcasses that showed evidence of bloody discharge from the anus, vulva, nostrils, mouth, eyes, or ears and incomplete rigor mortis. Microscopic examination of methylene blue–stained blood smears was conducted for some carcasses. Smears positive by microscopy (detection of encapsulated bacilli) were combined with probable cases (27% of probable cases were confirmed by microscopy) for analyses. No samples were confirmed by bacterial culture and isolation because of lack of facilities locally. Because of abundant scavengers in the ecosystem and logistical challenges of surveillance over such a large and remote area, many cases will go undetected on the basis of our definitions. Specifically, our definitions comprised only intact or partially scavenged carcasses; this second group also included carcasses obtained during major reported outbreaks. Carcasses that were too decomposed or scavenged to enable classification were not included in either group.

### Serum Samples

Serum samples were obtained opportunistically as part of long-term epidemiologic and ecologic studies. Samples were obtained from 3 groups. The first group comprised wild carnivore species, including 263 Serengeti and 23 Ngorongoro Crater lions obtained during 1985–2007 and 53 Serengeti spotted hyenas (*Crocuta crocuta*) obtained during 1998–2007. The second group comprised Serengeti and Ngorongoro Crater ungulate species (28 and 21 buffalo [*Syncerus caffer*], 36 and 23 wildebeest [*Connochaetes taurinus*], and 74 and 11 zebra [*Equus burchellii*], respectively) obtained during 1998–2007. The third group comprised domestic dog (*Canis familiaris*) populations living adjacent to protected areas (4 villages in Loliondo Division, 13 in NCA, and 7 from areas west of SNP), which included 169 random samples obtained as part of other epidemiologic surveys and 53 samples linked to a major anthrax outbreak in pastoralist areas in 2006.

### Serologic Assays

The QuickELISA Anthrax-PA Kit immunoassay (Immunetics Inc., Boston, MA, USA) was used to detect antibodies against protective antigen (PA) of *B. anthracis* in serum samples (*13*). The assay detects immunoglobulins in a subtype-independent and species-independent manner. The assay was conducted according to the manufacturer’s instructions. Briefly, serum (10 µL) was incubated with a mixture of 2 recombinant PA (rPA) conjugates: streptavidin-rPA and horseradish peroxidase–rPA. PA-specific, multivalent antibodies formed ternary complexes in which streptavidin-rPA and horseradish peroxidase–rPA were bound to different antigen-combining sites on 1 antibody molecule. Complexes were bound to the biotin-coated microplates by using streptavidin conjugate and detected by using horseradish peroxidase conjugate.

Bound antibodies against PA were detected by using a chromogenic peroxidase substrate (tetramethylbenzidine). The color development reaction was stopped by addition of 2N sulfuric acid. Absorbance at 450 nm, corrected by a 620–650 nm background subtraction, was measured by using an ELISA microplate reader. Interpretation was based on comparison of absorbance for the sample with an assay-defined cutoff value. The QuickELISA Anthrax-PA kit was configured to detect ≈300 ng/mL of PA-specific antibody at the cutoff value. The assay cutoff value was defined as the mean net absorbance at 450 nm plus 0.1 of the negative control (provided in the kit); the targeted cutoff range was 0.11–0.25.

### Data Analysis

A generalized linear modeling framework was used to investigate seroprevalence patterns in domestic dogs and wildlife with a binary outcome (seronegative or seropositive) and binomial error structure. Three models were constructed. First, we tested for overall species-specific differences in seroprevalence among lion, hyena, buffalo, wildebeest, and zebra populations in SNP and Ngorongoro Crater. Second, we tested for differences in seroprevalence in lion and hyena populations between years. Third, we analyzed seroprevalence patterns in domestic dogs in relation to their age and geographic location; village was modeled as a random effect by using a generalized linear mixed model.

## Results

### Case-Detection Patterns

#### Wildlife Cases

Potential anthrax cases, i.e., suspected and probable cases, were detected in a wide range of wildlife species, including wildebeest, buffalo, impala (*Aepyceros melampus*), giraffe (*Giraffa camelopardalis*), Thomson’s gazelle (*Eudorcas thomsonii*), and Grant’s gazelle (*Nanger granti*), hippopotamus (*Hippopotamus amphibius*), elephant (*Loxodonta africana*), and topi (*Damaliscus korrigum jimela*) (Table 1). The anthrax-attributed deaths of 1 cheetah (*Acinonyx jubatus*) and 1 serval cat (*Leptailurus serval*) during an outbreak in 1998 were the only carnivore cases reported during the study.

**Table 1:** Potential anthrax cases detected in Serengeti wildlife species, Tanzania, 1996–2009

Common name	Species	No. cases	
		Suspected	Probable
Baboon	*Papio anubis*	0	1
Black rhinoceros	*Diceros bicornis*	1	0
Buffalo	*Syncerus caffer*	20	85
Bushbuck	*Tragelaphus scriptus*	1	0
Cheetah	*Acinonyx jubatus*	0	1
Duiker	*Sylvicapra grimmia*	0	1
Eland	*Taurotragus oryx*	0	1*
Elephant	*Loxodonta africana*	24	7
Grant’s gazelle	*Nanger granti*	2	3
Giraffe	*Giraffa camelopardalis*	14	8
Hartebeest	*Alcelaphus buselaphus*	0	1
Hippopotamus	*Hippopotamus amphibious*	32	14
Impala	*Aepyceros melampus*	35	659
Ostrich	*Struthio camelus*	0	1
Serval	*Leptailurus serval*	0	1
Thomson’s gazelle	*Eudorcas thomsonii*	7	5
Topi	*Damaliscus korrigum jimela*	0	4
Vulture	*Gyps africanus*	0	2
Warthog	*Phacochoerus africanus*	0	2
Waterbuck	*Kobus ellipsiprymnus defassa*	1	1
Wildebeest	*Connochaetes taurinus*	112	60
Zebra	*Equus burchellii*	34	83

*Outbreak reported but individual case counts not available.

Cases were detected every year in NCA and SNP. However, major reported outbreaks were limited in time and space (Figure 1, panel A). Species affected and extent of outbreaks varied. Impalas were predominantly affected during 2 outbreaks (in southcentral Serengeti in late January–early February 1998 [*14*] and in central Serengeti in January 2003). Wildebeest and zebras were mostly affected during an outbreak east of SNP in early 2006. Buffalo were most recently affected southwest of SNP in October 2009. Small numbers of zebra deaths were recorded regularly throughout the study period, in addition to the 2006 outbreak.

#### Livestock Cases

**Figure 2:**
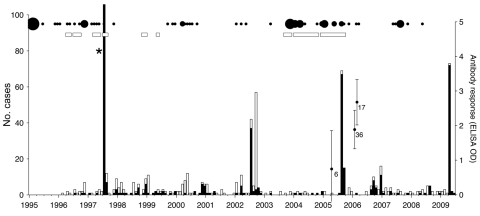
Anthrax case detection in wildlife, livestock, and human populations in the Serengeti ecosystem, Tanzania, 1996–2009. Probable (black bars) and suspected (white bars) wildlife cases (as defined in the Materials and Methods) are shown. Black circles indicate hospital records of anthrax scaled according to the number of cases, and rectangles indicate when cases in livestock were reported (quality of the data for livestock cases was too poor to quantify). Domestic dogs were sampled in villages near wildlife cases detected in 2006. Error bars indicate mean antibody responses and 95% confidence intervals at the time of sampling; sample sizes are indicated. *During the 1998 outbreak, 549 probable cases and 67 additional suspected cases were detected. OD, optical density.

During 1996–1999, several large outbreaks (>500 deaths) were documented in livestock (goats, sheep, and cattle) east of SNP. Suspected cases were reported regularly in some localities in apparently localized disease-endemic foci (i.e., Olbalbal, Oiti, and Olduvai; Figure 1, panel B). Small-scale vaccinations in the local vicinity were generally performed in response to these outbreaks. No livestock cases were reported from 2000 until the end of 2003, during which time considerable livestock vaccination was conducted. Livestock cases have been reported since 2004, and many livestock carcasses were obtained during the 2006 wildlife outbreak (Figure 2). However, local Maasai communities attributed these deaths to starvation, and diagnostic material was not available for confirmation.

#### Human Cases

Hospital records of anthrax-infected humans were typified by sporadic reports of nonfatal cutaneous anthrax from a few localities (Figure 1, panel A; Figure 2). However, small-scale household questionnaire surveys conducted in villages where livestock and wildlife anthrax outbreaks had occurred indicated several cases in persons who had eaten affected livestock carcasses (50% case-fatality rate, 4 deaths and 8 cases in 7,538 persons). Clinical signs included diarrhea and swollen abdomen (consistent with ascites), which are features of gastrointestinal anthrax.

### Serologic Patterns

#### Wildlife

**Figure 3:**
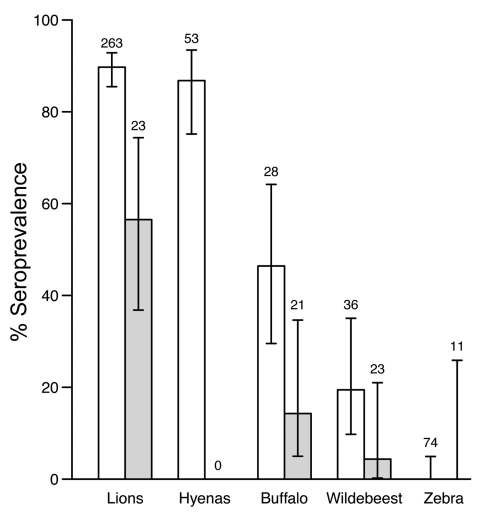
Seroprevalence of anthrax in sampled wildlife populations from Serengeti National Park (white bars) and Ngorongoro Crater (gray bars), Tanzania, 1996–2009. Error bars indicate 95% confidence intervals. Sample sizes used to calculate seroprevalence are indicated above the bars. Hyenas were not sampled in Ngorongoro Crater. Seropositive zebras were not detected; error bars indicate 95% confidence intervals based on a binomial distribution of the sample size and the seropositivity range that can be expected.

**Figure 4:**
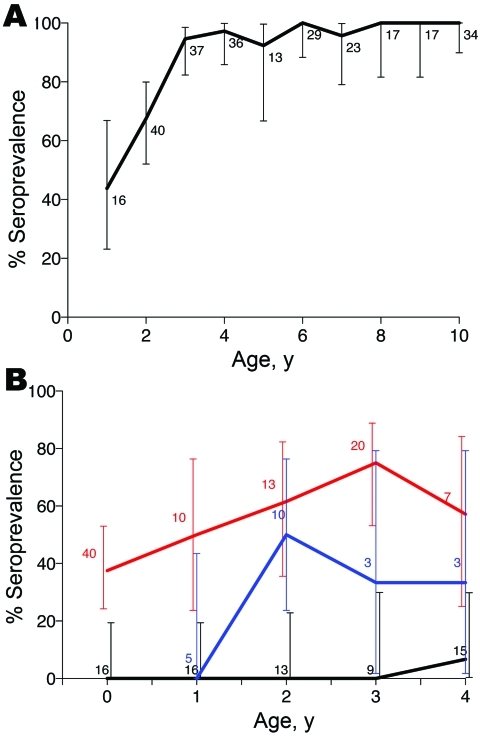
Anthrax seroprevalence patterns in carnivores, by age, Tanzania, 1996–2009. Lions (A) in Serengeti and domestic dogs (B) in agropastoralist regions where no outbreaks were detected (black line), in pastoralist regions where repeated outbreaks were detected (red line), and in an agropastoralist village where no outbreaks were reported but serologic surveys indicated previous exposure (blue line). Error bars indicate 95% confidence intervals for age seroprevalence in lions and dogs, but are juxtaposed for dogs to improve readability. Sample sizes used to calculate seroprevalences are indicated.

Seroprevalence patterns of sampled wildlife varied widely and showed differences among species and populations (Figure 3). Overall seroprevalence was lower in wildlife populations in Ngorongoro than in Serengeti (p<0.001). Seroprevalence was consistently high in wild carnivores (90% and 57% overall seropositivity in Serengeti and Ngorongoro Crater lions, respectively, and 87% seropositivity in Serengeti spotted hyenas) and did not show any significant year-to-year variation, e.g., in years of known outbreaks (p>0.05). Age seroprevalence in Serengeti lions indicated a high frequency of infection, seroconversion at a young age, and seropositive animals in all age groups (Figure 4, panel A). Seroprevalence was lower among herbivorous species (46% and 14% seropositivity in Serengeti and Ngorongoro Crater buffalo and 19% and 4% in Serengeti and Ngorongoro Crater wildebeest); no zebras were found to be seropositive despite relatively extensive sampling efforts (Figure 3).

#### Domestic Dogs

Seroprevalence patterns in domestic dog populations showed marked regional differences (Figure 1, panel B). Low seroprevalence was observed in agropastoralist western communities, and high and spatially variable seroprevalence was observed in pastoralist eastern communities (p<0.001) (Figure 1, panel B; Table 2). More specifically, high seroprevalence was recorded in dogs in pastoralist areas where livestock cases had been regularly reported (e.g., Olbalbal, Oiti, and Olduvai areas). High seroprevalence was also observed in domestic dogs sampled 6 weeks after the outbreak was detected in zebras and wildebeest in Ngorongoro in early 2006 (Figure 4, panel B), and changes in mean antibody levels in domestic dogs sampled before and after the outbreak also reflected recent exposure (Figure 2). Most dogs sampled in western areas of the Serengeti were seronegative, which is consistent with an absence of reports of anthrax cases in either the veterinary office or hospital records. However, seropositive dogs were detected in 1 village, Gibeshi, where anthrax had not been previously recorded (Figure 1, panel B). Age seroprevalence patterns of dogs sampled in 1999 indicated seropositivity only in dogs >1 year of age, which is consistent with exposure occurring in 1997 or 1998 (Figure 4, panel B).

**Table 2:** Multivariate generalized linear mixed model analysis of risk factors for anthrax seropositivity in dogs, Tanzania, 1996–2009*

Predictor	Unit	Estimate	SE	*z* score	p value	OR (95% CI)
Intercept		−6.224	1.275	−4.881	<0.001	NA
Age	Months	0.043	0.011	3.956	<0.001	1.044 (1.022–1.066)
Area	West	Referent	Referent	Referent	Referent	1
	Loliondo	4.178	1.696	2.463	0.014	65.21 (2.347–1,811.000)
	NCA	4.511	1.364	3.308	<0.001	90.97 (6.283–1,317.000)

*OR, odds ratio; CI, confidence interval; NA, not applicable; NCA, Ngorongoro Conservation Area.

## Discussion

We report wide variation in patterns of exposure to anthrax and deaths among wild and domestic animal species and populations of the Serengeti ecosystem. Serologic data also highlight the potential value of domestic dogs as indicator species for identifying high-risk areas of infection for livestock and human populations.

The QuickELISA Anthrax-PA kit was a convenient method for assessing seroprevalence in the multiple species examined in this study. The assay does not rely on species-specific or protein A/G conjugates to detect anthrax-specific antibodies and thus can detect any multivalent antibody in a sample. Previous studies that examined antibodies against anthrax in wildlife required unique conjugates specific for each species studied, which necessarily limited the number of species that could be examined (*10*). In our study, antibodies were measured in 6 species by using 1 assay. Thus, relative amounts of antibody present in each sample could be directly compared.

Lack of obvious clinical signs before death, inaccessibility of remote locations, decomposition, and hazardous carcasses all affect the quality of anthrax surveillance based on case detection. Despite concerted efforts to obtain samples from suspected cases, we recovered little diagnostic material for confirmation. However, more probable cases were identified on the basis of the appearance of carcasses. Laboratory results may not be conclusive even when diagnostic material is obtained. For example, when multiple blood slides were prepared from 1 buffalo carcass and tested blindly, the results were not consistently positive (only 2 of 6 slides were positive). Some outbreaks are likely to be missed even when probable case detection is used. This suggestion was confirmed by using serosurveillance data; in some agropastoralist areas where anthrax had not been reported, serologic analysis of domestic dogs indicated that the disease had been present.

In contrast to sampling of suspected carcasses, serosurveillance of living animals poses no risk for anthrax infection and therefore offers an opportunity for gaining a better understanding of anthrax epidemiology, particularly in relation to patterns of infection and risk factors for exposure, susceptibility, and death. Our serologic data highlight differences between species in exposure and death, which may be explained by behavior and ecology. However, we caution that although our study suggests great potential for the use of this assay for multiple species comparisons, validation of serologic responses across a range of vaccinated species (possibly using zoo collections) would provide more definitive verification of this proposition and should be prioritized.

Low mortality rates, combined with high seroprevalence rates (always >50% and approaching 90% in Serengeti populations), suggests that wild carnivores are regularly exposed to anthrax without apparent deaths. Although high mortality rates were reported for Kruger lions after periods of low anthrax incidence (*4**,**10*), high seroprevalence rates and low mortality rates are more commonly observed, which suggests a protective immune response presumably associated with more frequent exposure (*4**,**10*). Lions and hyenas may be exposed through consumption of infected prey, but domestic dogs may also be exposed when they scavenge infected carcasses (wildlife and livestock). Low seroprevalence rates and high mortality rates have been reported for cheetahs (*15**,**16*). These rates are consistent with the fact that cheetahs do not scavenge, and solitary hunting exposes them to fewer carcasses than group-hunting lions and hyenas. Although data from this study are limited, detection of probable anthrax cases in cheetah and serval is consistent with a higher susceptibility in these carnivore species.

Relatively lower seroprevalence rates and higher mortality rates for ungulates than for carnivores suggest less routine exposure, higher susceptibility, or both. Major differences in herbivore susceptibility are inferred from the wide variation in seroprevalence detected in species sharing the same grazing areas, and thus probable exposure patterns. Zebras appear to be highly susceptible; however, buffalo and wildebeest can clearly survive infection. Seroprevalence in buffalo was high (≈50%) compared with previous reports for herbivores (≈7%) (*10**,**17*). Comparing seroprevalence in more species would enable assessment of relative roles of exposure versus susceptibility in explaining variable species mortality patterns characteristic for anthrax.

The reported species differences have potential implications for serosurveillance. Among wildlife, carnivores are likely to be the most sensitive indicators of whether infection is present in an area, acting as bioaccumulators of infection through consumption of infected carcasses (*18*). However, because lions and hyenas seroconvert at a relatively young age, temporal patterns of exposure from age seroprevalence data are difficult to detect. Furthermore, hyenas are highly mobile in the Serengeti ecosystem (*19*), which reduces their utility for identifying specific high-risk areas. However, more detailed investigations of titer levels in relation to timing and location of anthrax outbreaks, including longitudinal studies of serial titers from known animals, could shed light on immunologic responses and enable more information to be obtained from serologic data.

These data suggest a possible utility of serosurveillance in buffalo, whose potential as indicator species has not been explored. Because ≈50% of Serengeti buffalo are seropositive for anthrax, these populations appear to provide a relatively sensitive indicator of the presence and prevalence of anthrax infection, e.g., major differences between populations in SNP and Ngorongoro Crater. Although serologic analysis of wildebeest detected these differences, seroprevalence in wildebeest was lower overall, and the wide-ranging migratory movements of the Serengeti herds limit the utility of these data for detecting spatial patterns. In comparison, buffalo herds range over relatively restricted areas, and serologic data can pinpoint high-risk areas. In many protected areas of Africa, buffalo are already routinely sampled for surveillance of diseases, such as rinderpest, bovine tuberculosis, and foot-and-mouth disease. We suggest that in areas where buffalo surveillance is ongoing, there is added value in using serum samples for monitoring anthrax exposure patterns. Serologic analysis of buffalo and analysis of environmental risk factors could also assist wildlife management strategies, e.g., risks associated with reintroductions of rhinoceros in different areas of the Serengeti, and identify priority areas for enhanced risk-based surveillance.

Domestic dogs have high potential value as indicators of human and livestock diseases (*1**,**18*). They are regularly exposed to a wide range of infections in disease-endemic areas; they are abundant and widely distributed, especially in developing countries; they are generally accessible for safe handling and sampling; they can be sampled at young ages, which enables reasonably accurate timing of outbreaks; and they live in close association with humans and livestock, which makes them good indicators of risk. In addition, vaccination campaigns present a cost-effective opportunity for obtaining large numbers of domestic dog samples (*18*). Consistent with these expectations, we have demonstrated that dogs in the Serengeti can be useful indicators of anthrax. They can be used to detect infection in an area, even when anthrax is not identified in other species; they reflect differences in infection prevalence in different areas; they can provide information about the timing of outbreaks (we observed variation in exposure with age) (Table 2); and they serve as an indicator of livestock and human disease risk and provide a basis for risk-based surveillance and targeted implementation of prevention measures (e.g., livestock vaccination or public health campaigns).

In conclusion, we demonstrated that serologic investigations of wildlife and domestic animals can provide valuable information about patterns of anthrax transmission and for identifying areas for risk-based surveillance. Serologic approaches also enable retrospective identification of infected areas and timing of outbreaks where case surveillance is limited because of remoteness of an area, poor reporting of cases to local or central authorities, misdiagnosis, and difficulties in performing confirmatory laboratory diagnostic tests. Further research may enable more effective use of serologic data if insights can be gained into how antibody levels relate to timing and degree of exposure.
